# Is Automatic Tumor Segmentation on Whole-Body ^18^F-FDG PET Images a Clinical Reality?

**DOI:** 10.2967/jnumed.123.267183

**Published:** 2024-07

**Authors:** Lalith Kumar Shiyam Sundar, Thomas Beyer

**Affiliations:** Quantitative Imaging and Medical Physics Team, Medical University of Vienna, Vienna, Austria

**Keywords:** image processing, research methods, AI, tumor segmentation, whole-body PET

## Abstract

The integration of automated whole-body tumor segmentation using ^18^F-FDG PET/CT images represents a pivotal shift in oncologic diagnostics, enhancing the precision and efficiency of tumor burden assessment. This editorial examines the transition toward automation, propelled by advancements in artificial intelligence, notably through deep learning techniques. We highlight the current availability of commercial tools and the academic efforts that have set the stage for these developments. Further, we comment on the challenges of data diversity, validation needs, and regulatory barriers. The role of metabolic tumor volume and total lesion glycolysis as vital metrics in cancer management underscores the significance of this evaluation. Despite promising progress, we call for increased collaboration across academia, clinical users, and industry to better realize the clinical benefits of automated segmentation, thus helping to streamline workflows and improve patient outcomes in oncology.

In the domain of oncologic imaging, ^18^F-FDG PET/CT has established itself as an indispensable tool facilitating the detection and management of tumors through the visualization of metabolic activity. By highlighting areas of increased glucose consumption, this imaging technique enables clinicians to discern malignancies from benign tissues, thus providing key information to the diagnosis, staging, and evaluation of therapeutic response in cancer patients.

The quantification of tumor burden through metabolic tumor volume, disease dissemination index, and total lesion glycolysis has further refined the utility of ^18^F-FDG PET, offering prognostic value and aiding in the stratification of treatment approaches (1–5). These parameters encapsulate both the volume and the metabolic intensity of tumors and thereby serve as crucial indicators of tumor aggressiveness and response to therapy. However, the manual segmentation of tumor volumes is labor-intensive and subject to interobserver variability, hence limiting its feasibility in routine clinical practice. In response to these challenges, and in view of the ever-increasing workload, there is renewed interest in automatic whole-body tumor ^18^F-FDG volume segmentation so as to enhance the reproducibility and efficiency of tumor burden assessments.

## DIGITAL DISSECTION: ACADEMIA BETS ON AI FOR TUMOR SEGMENTATION

Today, the academic community has shifted attention toward leveraging artificial intelligence (AI) for multiple tasks along the imaging value chain, most notably perhaps toward automated whole-body PET ^18^F-FDG tumor segmentation. This transition seeks to surmount the limitations inherent in traditional thresholding techniques, which often indiscriminately encapsulate both physiologic and pathologic tissues.

By harnessing the sophistication of AI algorithms—particularly those evolving from the foundational U-Net architecture—there is a concerted effort to precisely target and segment pathologic tissues, without the inclusion of nonpathologic regions. Initiatives such as AutoPET (6) and HECKTOR (7) have been pivotal, providing open-source datasets that are instrumental for the training and refinement of AI models. Among the array of methodologies used, nnU-Net (8) and MONAI’s Auto3Dseg (9) stand out, by offering robust performance for enhanced accuracy in tumor segmentation building on a wealth of curated training datasets.

Despite the body of academic publications that show success and advocate for AI-driven tumor segmentation methodologies, there is a notable paucity of open-source solutions, a gap that poses significant challenges to the advancement of the imaging field. Moreover, the available open-source datasets are predominantly focused on specific types of cancers—namely lymphoma, lung cancer, and melanoma for AutoPET (6) and head and neck cancer for HECKTOR (7). This specialization limits their utility for training AI models that are generalizable across a broader spectrum of cancers and centers.

## SMART SIMPLICITY: COMMERCIAL TOOLS ARE OFFERING PRACTICAL SOLUTIONS

In response to the market need for automated lesion segmentation tools, leading commercial vendors make significant strides in the development of fully automated and semiautomated methodologies for whole-body tumor volume segmentation from ^18^F-FDG PET images. These methodologies commence with threshold-based segmentation to highlight hypermetabolic regions, which inherently include both pathologic and physiologic tissues. The challenge then is in distinguishing these tissues accurately, a task that vendors are addressing with distinct strategies.

For example, Auto ID (Siemens Healthineers) uses AI to differentiate between pathologic and physiologic tissues after an initial threshold-based segmentation (1). This technique signifies a step toward full automation, aiming to reduce the need for manual intervention for ensuring segmentation accuracy. In contrast, Hermes Medical Solutions and MIM Medical have embraced a simplified, semiautomatic, one-click methodology. They have introduced Single Click Segmentation (Hermes) and Lesion ID (MIM), which allow medical professionals to easily refine the prethresholded segmentations with just one click, effectively isolating nonpathologic tissues. Essentially, this approach involves users manually identifying and categorizing regions within the prethresholded segmentation as either nonpathologic or pathologic. This user-friendly approach emphasizes simplicity while providing clinicians with straightforward segmented regions for clinical use. By seeking to provide certified tools that balance efficiency with practicality, commercial vendors are playing a pivotal role in the ongoing effort to improve diagnostic processes and, ultimately, patient outcomes in the field of oncology.

## FROM BENCH TO BEDSIDE: HAS AUTOMATED VOLUME PARAMETER EXTRACTION PROLIFERATED TO CLINICS?

As discussed earlier, extensive clinical research has underscored the significance of volumetric parameters from ^18^F-FDG PET/CT, specifically metabolic tumor volume and total lesion glycolysis, in enhancing prognostic evaluations and monitoring therapeutic responses across a diverse spectrum of cancers. Despite the significant potential of both parameters, they are not being used in routine clinical practice or trials. However, on the basis of our personal correspondence with both nuclear medicine clinicians and vendors, there seems to be a growing interest from the clinical community to extract volume-based metabolic parameters for lymphoma patient management (4,5). There is also an expectation toward fully automated solutions, as manual corrections can be tedious in patients with extensive disease.

## THE FINAL FRONTIER: COMPLETE AUTOMATION—POSSIBLE OR PREPOSTEROUS?

Complete automation of target region segmentation might be possible with AI. For example, fully automatic, CT-based organ segmentation is now a reality with strong open-source solutions (10,11). Both academic and industrial sectors show unanimous interest in harnessing AI methodologies for PET-based tumor segmentation. This convergence of interest, however, encounters notable challenges, particularly with the generalizability of AI models across various cancer types. The capability of an AI algorithm, trained on ^18^F-FDG PET/CT images for lung cancer segmentation, to perform equally well on colorectal cancer, for instance, remains in question. There is initial evidence suggesting that algorithms designed for lung cancer might be adaptable for breast cancer segmentation when ^18^F-FDG PET/CT images are used (12). Nonetheless, the issue of algorithm generalizability is not confined to cancer types alone; it extends to differing imaging systems and reconstruction protocols across sites, further complicating the model’s adaptability (13).

The challenges of model generalizability are further amplified when considering the use of different imaging tracers. Nuclear medicine uses a broad spectrum of tracers to detect and quantify tumor characteristics, necessitating the development of distinct AI models for each tracer. This requirement imposes an economic burden, as clinics face escalating costs with the introduction of each new model by vendors (14). Such a situation underscores a significant shortcoming in the current methodologic approach, highlighting the urgent need for economically viable and universally applicable AI solutions. Moreover, the extensive and varied nature of these challenges underscores the critical need for comprehensive, large-scale validation studies. These studies are indispensable for affirming the preliminary evidence and for assessing the real-world applicability of AI algorithms across different cancers, imaging tracers, and health care settings.

In light of current trends in AI, the ideal solution appears to be the development of a large, unified foundational tool capable of segmenting various tracer images. However, achieving regulatory approval for such a tool is challenging because of the specificity of intended uses outlined in certification processes. Furthermore, the regulatory landscape for AI applications in health care in general is fraught with seemingly high barriers in the complex process of validation and certification.

In addition, the availability of comprehensive and well-curated PET datasets remains limited, a surprising fact given the modality’s long-standing presence. In contrast, the field of radiology has seen significant advancements through the open sourcing of its datasets while effectively addressing privacy concerns (15). Therefore, there is a pressing need to create extensive databases of PET images and to secure funding for expert labeling or to engage labeling services. Recent developments in advanced vision foundational models, such as the segment anything model (SAM) (16), offer promising solutions by enabling segmentation through points, bounding boxes, or prompts. These models are already being explored in medical imaging, with significant investment from commercial vendors, such as United Imaging Healthcare for clinical applications (17,18).

Despite many methodologic and regulatory hurdles, the potential benefits of offering the prospect of a single, versatile tool capable of segmenting any tissue of interest are immense. To ensure the seamless integration of these methodologies into clinical workflows, it is imperative that they enhance clinical workflows without adding complexity or significant cost. This approach not only fosters innovation but also encourages a harmonious blend of technology and clinical practice for the betterment of patient care.

## STATUS QUO AND STATUS GO

The beginning integration of AI into oncologic imaging, particularly with ^18^F-FDG PET/CT, marks a significant step forward in the management and treatment of cancer. Although AI promises to streamline diagnostic processes and improve accuracy, hurdles such as model generalizability, economic viability, and legislative barriers pose significant challenges to AI’s broader application for automated tumor segmentation in the clinic. The path forward necessitates collaborative research, increased funding, and the creation of extensive PET image databases. These measures are vital to advance AI methodologies to a level where they can be effortlessly integrated into clinical practices without burdening medical professionals. In addition, targeted grant support for labeling services is crucial to enhance the accuracy and effectiveness of AI models across different cancer types.

Current PET-based AI algorithm development for tumor segmentation places a significant emphasis on optimizing metrics such as the Dice similarity coefficient (DSC). For instance, leaderboards such as AUTOPET (6) primarily highlight DSC scores, with top reported values of around 0.37. However, initiatives such as HECKTOR (7) extend their focus beyond DSC (top DSC, 0.79), incorporating both prediction accuracy and DSC to evaluate algorithms, acknowledging the need for algorithms to predict clinical outcomes such as overall survival, progression-free survival, or treatment response. Although achieving high DSCs is commendable for technical precision, such as in CT organ segmentation, it may not suffice for the clinical applicability required in PET tumor segmentation. For example, a DSC of 0.70 could offer prognostic accuracy comparable to manual segmentation, suggesting that beyond a certain threshold, further technical advancements might not result in significant clinical improvement. This situation calls for a strategic shift in research priorities, aiming to identify the minimum accuracy threshold that meaningfully enhances clinical endpoints, thereby ensuring that algorithm development aligns with clinical needs and contributes effectively to patient management.

As we go forward, success in overcoming the complex challenge of tumor segmentation relies on collective effort rather than solitary endeavors. The solution extends beyond the capacity of any single entity, requiring a collaborative approach that leverages the strengths of academia, industry, and clinical practitioners. Clinicians, in their unique role, are invited to articulate key requirements for their expertise to be ventilated by technical and methodologic progress; likewise, they are also instrumental in initiating and contributing to large-scale, open-source databases that have high-quality data with appropriate metadata (cancer type, stages, imaging systems, reconstruction protocol, etc.) and annotations, thus laying the groundwork for developing precise AI models. The importance of standardized annotations for generating high-quality datasets cannot be overstated. Therefore, it is imperative that clinicians define and adopt consensus guidelines during the annotation process (19).

Academia can contribute through rapid innovation, developing open-source low-click annotation tools (e.g., MedSAM (18) and MONAILabel (20)) and pioneering segmentation technologies to keep pace with the evolving demands of tumor segmentation. Recent academic efforts have highlighted the role of hyperparameter changes, minor architecture adjustments, and data augmentation in improving the accuracy of the tumor segmentation (21,22). Finally, industry can augment these efforts by providing essential resources through research funding support and applying business expertise to ensure that the promising innovations are practically and sustainably deployed in real-world scenarios.

We believe there is a significant opportunity for both academic institutions and businesses to collaborate more closely. This collaboration via research agreements could extend beyond data sharing or independently evaluating the solutions offered by industry. For instance, they could work together on creating generic software frameworks that would serve the interests and needs of both the academic and the industrial sectors. An example of such successful collaboration is the MONAI framework (9), cocreated by Nvidia and King’s College London, which demonstrates how these partnerships can yield durable solutions that serve both research and clinical needs.

The primary issue with academic software is its transient nature and the maintenance challenges it faces, largely due to a lack of incentives for ongoing support. Collaborations with industry not only aim to address this issue but also provide academics with crucial experience in developing sustainable software. This symbiotic relationship fosters an environment for innovation, allowing academia to translate research into practical applications and allowing industry to identify and cultivate technologic advancements that are appropriate for clinical use. However, for these partnerships to thrive, it is imperative to define rules of engagement and roles, intellectual property rights, and monetization strategies from the start. The benefits of such collaborations—ranging from accelerated technologic progress and improved software sustainability to ultimately better patient care—underscore their importance for the future of AI applications in nuclear medicine.

## DISCLOSURE

No potential conflict of interest relevant to this article was reported.
